# GGTA1/iGb3S Double Knockout Mice: Immunological Properties and Immunogenicity Response to Xenogeneic Bone Matrix

**DOI:** 10.1155/2020/9680474

**Published:** 2020-06-03

**Authors:** Anliang Shao, You Ling, Liang Chen, Lina Wei, Changfa Fan, Dan Lei, Liming Xu, Chengbin Wang

**Affiliations:** ^1^Medical School of Chinese PLA & Medical Laboratory Center, First Medical Center of Chinese PLA General Hospital, Beijing 100853, China; ^2^Institute for Medical Device Control, National Institutes for Food and Drug Control, Beijing 102629, China; ^3^National Engineering Laboratory for Regenerative Medical Implant Devices, Guanhao Biotech, Co., Ltd., Guangzhou 510530, China; ^4^Institute for Laboratory Animal Resources, National Institutes for Food and Drug Control, Beijing 102629, China

## Abstract

**Background:**

Animal tissues and tissue-derived biomaterials are widely used in the field of xenotransplantation and regenerative medicine. A potential immunogenic risk that affects the safety and effectiveness of xenografts is the presence of remnant *α*-Gal antigen (synthesized by *GGTA1* or/and *iGb3S*). *GGTA1* knockout mice have been developed as a suitable model for the analysis of anti-Gal antibody-mediated immunogenicity. However, we are yet to establish whether *GGTA1/iGb3S* double knockout (G/i DKO) mice are sensitive to Gal antigen-positive xenoimplants.

**Methods:**

*α*-Gal antigen expression in the main organs of G/i DKO mice or bovine bone substitutes was detected via a standardized ELISA inhibition assay. Serum anti-*α*-Gal antibody titers of G/i DKO mice after immunization with rabbit red blood cells (RRBC) and implantation of raw lyophilized bone substitutes (Gal antigen content was 8.14 ± 3.17 × 10^12^/mg) or Guanhao Biotech bone substitutes (50% decrease in Gal antigen relative to the raw material) were assessed. The evaluation of total serum antibody, inflammatory cytokine, and splenic lymphocyte subtype populations and the histological analysis of implants and thymus were performed to systematically assess the immune response caused by bovine bone substitutes and bone substitute grafts in *G/i* DKO mice.

**Results:**

*α*-Gal epitope expression was reduced by 100% in the main organs of G/i DKO mice, compared with their wild-type counterparts. Following immunization with RRBC, serum anti-Gal antibody titers of G/i DKO mice increased from 80- to 180-fold. After subcutaneous implantation of raw lyophilized bone substitutes and Guanhao Biotech bone substitutes into G/i DKO mice, specific anti-*α*-Gal IgG, anti-*α*-Gal IgM, and related inflammatory factors (IFN-*γ* and IL-6) were significantly increased in the raw lyophilized bone substitute group but showed limited changes in the Guanhao Biotech bone substitute group, compared with the control.

**Conclusion:**

G/i DKO mice are sensitive to Gal antigen-positive xenogeneic grafts and can be effectively utilized for evaluating the *α*-Gal-mediated immunogenic risk of xenogeneic grafts.

## 1. Introduction

Animal tissue-derived biomaterials are widely used in the field of tissue engineering and regenerative medicine. All mammalian species except humans, apes, and Old World monkeys produce the Gal *α*1,3-Gal*β*1-4GlcNAc-R (*α*-Gal) antigen [1], due to *α*-Gal gene being inactivated as a result of frame shift and nonsense mutations [2, 3]. However, continuous antigenic stimulation by the gastrointestinal flora results in the production of ~1–3% of natural anti-Gal Ab in these primates [4–6]. Natural anti-Gal Ab are responsible for hyperacute rejection (HAR) in pig-to-primate xenotransplantation [7, 8]. Undesirable immunological responses triggered by remnant *α*-Gal epitope in biomaterials remain a significant problem that affects tissue regeneration and remodeling [9, 10]. Therefore, using wild-type (WT) animals to evaluate immunogenicity caused by remnant *α*-Gal antigen in animal tissue-derived biomaterials is not feasible since WT animals express *α*-Gal antigen, but not humans.

The *α*-Gal antigen is synthesized by *α*1,3 galactosyltransferase 1 (*GGTA1*) and isoglobotrihexosylceramide 3 synthase (*iGb3S*) [11–13]. The binding properties of anti-Gal antibodies from *GGTA1* knockout (*GGTA1* KO) mice to *α*-Gal antigen are reported to be similar to those of human anti-Gal antibodies. Furthermore, *GGTA1* KO mice developed acute immune rejection after implantation of xenogenic cardiac patches containing *α*-Gal antigen [14]. A reduction in *α*-Gal l antigen of a porcine xenobone graft led to significant suppression of the humoral immune response to *α*-Gal antigen in C57BL/6 *α*-Gal knockout mice and consequent improvement in histologic union [15]. The highest levels of IgM and IgG antibodies were detected in fresh porcine posterior corneal lamellar grafts in *GGTA1* KO mice while reduced IgG deposition was observed in fresh porcine posterior corneal lamellar grafts in WT mice and decellularized porcine posterior corneal lamellar grafts in *GGTA1* KO mice [16]. Our previous study showed that implantation of raw lyophilized bone substitutes into *GGTA1* KO mice induced a significant increase in anti-*α*-Gal antibody and Th2 immune reaction-related inflammatory factors while *α*-Gal antigen-decreased bone substitutes (Guanhao Biotech bone substitutes) displayed slight changes relative to the control [17]. These findings support the suitability of *GGTA1* KO mice as a model for the analysis of anti-Gal antibody-mediated immunogenicity.

Several studies have shown that *iGb3S* plays a role in *α*-Gal epitope expression [11, 18, 19]. Previously, we demonstrated that *iGb3S* gene knockout induced an ~5.19–21.74% decrease in *α*-Gal epitope expression in main organs while deletion of *iGb3S* alone did not cause significant changes in the immunological properties of *iGb3S* KO mice with exogenous Gal antigen (rabbit red blood cells (RRBC)) stimulation [20]. Currently, it remains to be unknown whether *α*-Gal antigen expression in *GGTA1/iGb3S* double knockout (G/i DKO) animals is completely eliminated. Moreover, compared to *GGTA1 KO* mice, the sensitivity of G/i DKO mice to the stimulation of animal tissue-derived biomaterials whether improved is yet to be determined.

In the present study, to identify a suitable animal model for the immune risk assessment of animal-derived biomaterials, G/i DKO mice were generated. *α*-Gal epitope expression profiling in the main organs was performed, and immunogenic properties of the novel G/i DKO mouse model were assessed. The immunogenic responses of G/i DKO mice to raw lyophilized bone substitutes and Guanhao Biotech bone substitutes were evaluated. Furthermore, sensitivity of G/i DKO mice to bone substitutes relative to *GGTA1* KO mice generated in our previous study was compared.

## 2. Materials and Methods

### 2.1. Animals and Materials

G/i DKO mice were generated with the patent of the National Institutes for Food and Drug Control (China, ZL201510122581.1). Briefly, *GGTA1* and *iGb3S* genes were isolated from genomic DNA, homologous arms were obtained by amplification by long chain PCR method, and target vectors were constructed by combining with antibiotic resistance genes. The target carrier was transferred into the embryonic cells, and the recombinant embryonic cells were injected into the embryos of surrogacy animals and transplanted into the pseudopregnant animals to mate with normal animals. Genotype verification was carried out on the obtained chimera animals; then the genes successfully knocked out were screened. Chimera animals were mated with wild-type animals to obtain heterozygotes of F1 generation; the heterozygotes of F1 generation were mated with each other to become homozygous with both chromosomes removed. Then, the *GGTA1* and *iGb3S* homozygous animals were mated to screen the homozygous animals with both *GGTA1* and *iGb3S* deletion, and the double gene knockout animal population was obtained.

C57BL/6 wild-type (WT) mice were provided by the Institute for Laboratory Animal Resources/National Institutes for Food and Drug Control (NIFDC, China). Rabbit red blood cells were collected from New Zealand white rabbits (SPF grade). All animals were maintained in a specific pathogen-free facility under the following conditions: 23 ± 1°C, relative humidity of 30–70%, and 12 h light/12 h dark cycle. Animals were housed and handled in accordance with the guidelines set by the Association for the Assessment and Accreditation of Laboratory Animal Care. The study was approved by the NIFDC Institutional Animal Care and Use Committee (No. 2019A099).

Raw lyophilized bone substitutes (raw material) and Guanhao Biotech bone substitutes (medical products for bone repair) were provided by Guanhao Biotech Co., Ltd. (Guangzhou, China)

### 2.2. *α*-Gal Antigen Determination

*α*-Gal antigen expression in the heart, liver, spleen, lung, and kidney of experimental mice (WT: 13 weeks old, *n* = 6; DKO: 13 weeks old, *n* = 6) was determined via a standardized ELISA inhibition assay using a commercial *α*-Gal antigen detection kit (Meitan 70101, Beijing Sanyao Science & Technology Development Co., Beijing, China) according to our previous study [21]. Briefly, a calibration curve was produced using Gal-BSA (NGP0203, Dextra Laboratories, Redding, UK) combined with Gal-free matrix (380001–201701, Gal antigen-negative biomaterial reference material provided by NIFDC, Beijing, China) as a Gal antigen quantitative curve reference material. Gal antigen-positive and Gal antigen-negative biomaterial reference materials (380001–201701, provided by NIFDC, China) were used as the positive and negative controls, respectively, to monitor the sensitivity and specificity of the test system.

### 2.3. Immunization Treatment

Eight week-old G/i DKO mice (*n* = 12) were immunized with the RRBC membrane (1 × 10^8^ RRBCs) via intraperitoneal injection once (*n* = 6) or twice (*n* = 6) after a two-week interval. Blood samples from all animals were collected one week after the last treatment. Nontreated G/i DKO mice (*n* = 6) were used for comparative analyses.

### 2.4. Detection of Serum Anti-Gal Antibody after RRBC Immunization or Implantation

Serum levels of anti-Gal Ab in G/i DKO mice after RRBC immunization (anti-Gal IgG, IgM) or implantation (anti-Gal IgG, IgM and IgA) were detected via enzyme-linked immunosorbent assay (ELISA) using Gal-BSA (2.0 *μ*g/mL in carbonate buffer, pH 9.5) as a solid phase antigen. Briefly, 100 *μ*L/well Gal-BSA was loaded onto a 96-well plate for overnight coating followed by washing with PBS/0.05% Tween-20. The plate was blocked with 1% human serum albumin (HAS, A8230; Solarbio, Beijing, China) for 2 h at 37°C. Following a wash step, serial dilutions of each serum sample were generated in PBS/1%HAS and added to precoated plates. Plates were incubated for 2 h at 37°C and washed three times with PBS/0.05% Tween-20. Next, 100 *μ*L goat anti-mouse IgG (1 : 32,000) (IgG-HRP, sc-2005; Santa Cruz, CA, USA), IgM (1 : 16,000) (IgM-HRP, sc-2064; Santa Cruz, CA, USA), or IgA (1 : 1,000) (IgA–HRP, sc-3793; Santa Cruz, CA, USA) was added into each well for 1 h at 37°C. After washing, TMB (SE1005, Biokorad, Beijing, China) was added. Colorization was terminated by the addition of 10% H_2_SO_4_ and the optical density (OD) value at 450 nm determined using a microplate reader.

### 2.5. Preparation of Bone Substitutes

Raw lyophilized bone substitutes (T1) were prepared via physical cutting, freeze-drying, and sterilization from fresh bovine bone. Guanhao biotech bone substitutes (T2) were prepared from fresh bovine bone raw materials by physical cutting, decellularization, defatting, antigen removal, fixation, freeze-drying, and sterilization. The characterization and Gal antigen content determination of bone substitutes have been previously reported by our group [17].

### 2.6. Sample Implantation

Thirteen-week old G/i DKO mice (54 in total), including 27 female mice weighing 20.8 ± 1.6 g and 27 male mice weighing 27.2 ± 3.2 g, were divided into three groups (*n* = 18 per group, 9 females and 9 males) including three implantation periods (*n* = 6 per group at one implantation period, 3 females and 3 males). Raw lyophilized bone substitutes (set as T1), Guanhao biotech bone substitutes (set as T2), and false operation control (negative control group). Before implantation, all animals were anesthetized using 1.5% pentobarbital sodium (50 mg/kg). Samples (0.1 g) were implanted subcutaneously in the dorsal area while false operation was performed in the negative control group, which involved saline solution implantation. Blood samples from all groups (*n* = 6 per group, 3 females and 3 males) were collected for immune response evaluation at 2 weeks, 4 weeks, and 3 months after implantation, respectively. Spleen, thymus, and local implant tissues, including skin and implanted bone substitutes, were additionally harvested at these time-points after implantation for immune reaction test and pathological analysis.

### 2.7. Detection of Total Serum IgG, IgM, and IgA

Total serum IgG, IgM, and IgA contents of mice after implantation in the three groups were assessed via ELISA according to the manufacturer's protocol. Total serum IgG was detected using a mouse IgG (Total) ELISA Kit (EMC116, Neobioscience, Beijing, China) and serum samples diluted (1 : 4 × 10^6^). Total serum IgM was detected using an IgM Mouse ELISA Kit (88–50470-22, Affymetrix, Thermo Fisher Scientific, Massachusetts, USA) and serum samples diluted (1 : 6.4 × 10^6^). Total serum IgA was detected using a Mouse ELISA Kit (88–50470-77, Affymetrix, Thermo Fisher Scientific, Massachusetts, USA), and serum samples were diluted (1 : 2 × 10^5^). The optical density (OD) value was read at 450 nm using a microplate reader (Spectramax M5, Molecular Devices, CA, USA).

### 2.8. Serum Cytokine Measurements

The serum levels of soluble cytokines, including IFN-*γ*, IL-1*β*, and IL-6, were determined via Cytometric Bead Array analysis (CBA, BD Biosciences, New York, USA) according to the protocol provided by the manufacturer. Cytokine concentrations were obtained by converting mean fluorescence intensity using the standard curve [22].

### 2.9. Splenic Lymphocyte Subtype Analysis

The spleen was carefully dissected after mice were sacrificed at 2 weeks, 4 weeks, and 3 months. Spleen tissue was minced, homogenized, and filtered through a 70 *μ*m cell strainer. The cell suspension was centrifuged at 300 g for 5 min. Centrifugal precipitates were added to 1640 cell culture medium and recentrifuged under the same conditions to obtain splenic mononuclear cells. Mononuclear cells were suspended in 1640 cell medium at a concentration of 1 × 10^7^ cells/liter.

Labeled antibodies (FITC, anti-mouse CD3/PE, anti-mouse CD8a/PE, anti-mouse CD69/PerCP, anti-mouse CD45/APC, anti-mouse CD19/APC, and anti-mouse CD49b) were added to each flow tube. A splenic cell suspension of each sample (100 *μ*L) with a reasonable concentration was added to the flow tube. After thorough mixing, the reaction was conducted at room temperature for 20 min in the dark. Next, the mixture was washed with 2 mL PBS and centrifuged at 300 g for 5 min, following which the supernatant was discarded. After resuspension of the pellet in 0.5 mL PBS, cells were detected via flow cytometry (BD, LSR II, New York, USA).

### 2.10. HE Staining of Thymus and Implanted Local Tissue

Histologic analysis of thymus and implants, including implanted bone substitutes and subcutaneous surrounding tissues from all three groups, was performed using hematoxylin and eosin (HE) staining. Briefly, implants and thymus tissues were dissected and fixed with 10% buffered formalin solution for 48 h. Samples were subsequently dehydrated using alcohol and xylene and embedded in paraffin wax. All specimens were sliced into 5 *μ*m thick sections and stained with hematoxylin and eosin. Stained slides were examined under a light microscope.

### 2.11. Statistical Analysis

Numerical data from at least three individual experiments are presented as means ± SD unless otherwise indicated. Data were analyzed via one-way analysis of variance (ANOVA, Tukey's post hoc analysis) using statistical package SPSS 20.0 (SPSS Inc., Chicago, IL, USA). Statistical significance was set at ^∗^*p* < 0.05 versus the indicated group.

## 3. Results

### 3.1. *α*-Gal Antigen Content

In WT mice, *α*-Gal epitope expression was about 3- to 12-fold higher in the spleen and lung, compared with the heart, liver, and kidney (Table 1). In selected tissues of G/i DKO mice, *α*-Gal epitope expression was depleted by 100%, compared with WT mice (Table 1).

### 3.2. Serum Anti-Gal Antibody Levels after RRBC Immunization

**Figure 1:**
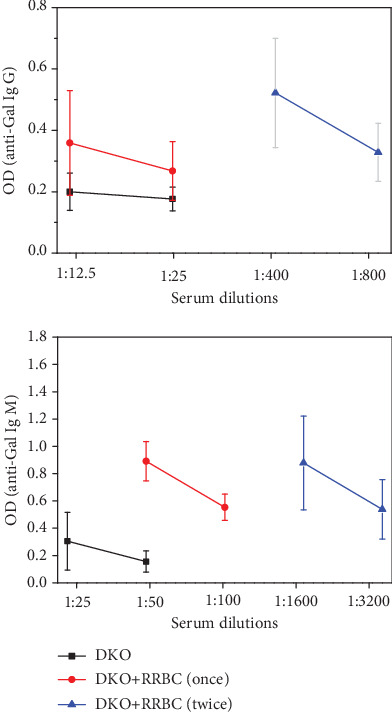
Serum anti-Gal antibody titers in G/i DKO mice with or without RRBC immunized. DKO: G/i DKO mice without RRBC stimulation; DKO+RRBC (once): G/i DKO mice with RRBC stimulation once; DKO+RRBC (twice): G/i DKO mice with twice RRBC stimulation.

Anti-Gal antibody titers of G/i DKO mice immunized once with RRBC were increased 5-fold for anti-Gal IgM while we observed no significant increase in anti-Gal IgG. After immunization with RRBC twice, anti-Gal antibody titers of G/i DKO mice were increased 80-fold for anti-Gal IgG and 180-fold for anti-Gal IgM (Figure 1).

### 3.3. Total Serum IgG, IgM, and IgA Levels

**Figure 2:**
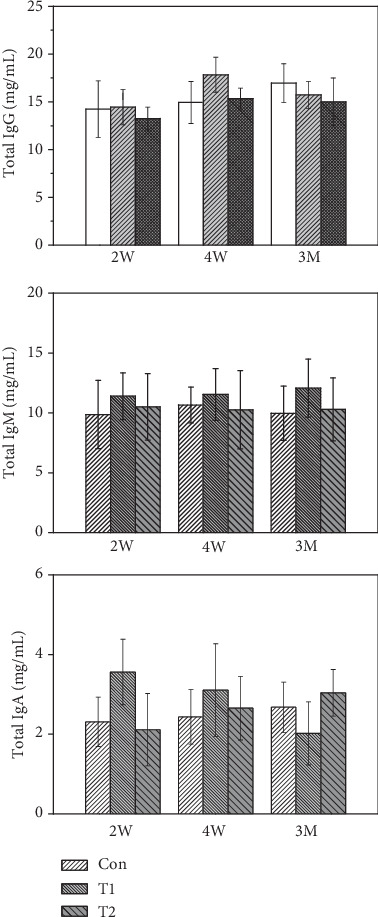
The total serum levels of IgG, IgM, and IgA from T1, T2, and control.

After implantation of bone substitutes at 2 weeks (2 W), 4 weeks (4 W), and 3 months (3 M), total serum levels of IgG, IgM, and IgA from the three groups were measured. Data are presented in Figure 2. At all three implantation times, total IgG, IgM, and IgA levels were comparable to those of the control.

### 3.4. Serum Anti-Gal Antibody Levels after Implantation

**Figure 3:**
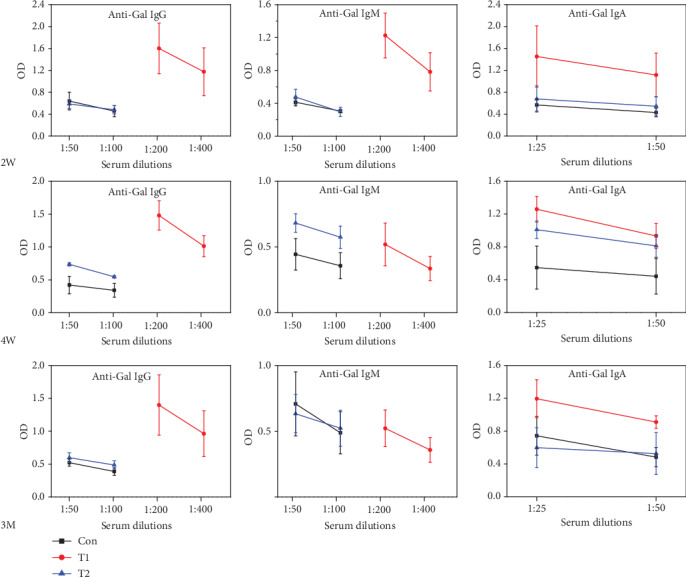
The IgG, IgM, and IgA anti-Gal antibody levels from T1, T2, and control.

Changes in the anti-Gal antibody levels are shown in Figure 3. At 2 W postimplantation, anti-Gal IgG and IgM levels of mice in the T1 group were about 15x higher while the anti-Gal IgA content was 3x higher than that in the control. However, no significant differences were observed in the IgG, IgM, and IgA anti-Gal antibody levels of mice in the T2 group, compared to the control.

At 4 W postimplantation, anti-Gal IgG, IgM, and IgA levels of mice in the T1 group were ~19x, 3x, and 1x higher relative to the control group, respectively. In contrast, no significant differences were observed in IgG and IgM anti-Gal antibody levels between the T2 and control groups. However, the anti-Gal IgA level of mice in the T2 group was 1x higher than that in the control.

At 3 M postimplantation, IgG, IgM, and IgA anti-Gal antibody levels of mice in the T1 group were ~15x, 1x, and 1x higher relative to the control, respectively. However, no marked changes happened in IgG, IgM, and IgA antibody levels between the T2 and control.

### 3.5. Serum Cytokine Levels

**Figure 4:**
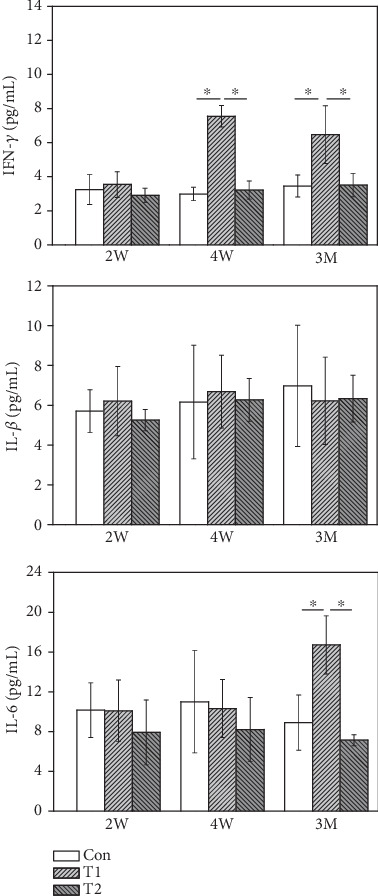
The serum cytokine levels of IFN-*γ*, IL-1*β*, and IL-6 level from T1, T2, and control.

The levels of the serum cytokines (IFN-*γ*, IL-1*β*, and IL-6) were determined to evaluate the immune responses triggered by T1 and T2 bone substitutes (Figure 4). The IFN-*γ* levels in the two bone substitute groups were comparable to that of the control at 2 W postimplantation. However, the IFN-*γ* level in T1 group was significantly higher than that in the control at 4 W and 3 M postimplantation while no differences were evident between the T2 and control. IL-1*β* levels in the two bone substitute groups were not significantly different relative to the control group at 2 W, 4 W, and 3 M postimplantation. Compared to the control, IL-6 levels of mice in the two bone substitute groups showed no changes at 2 W and 4 W postimplantation. Notably, the T1 group exhibited significantly higher IL-6 levels than control at 3 M postimplantation while no differences were observed between the T2 and control.

### 3.6. Splenic Lymphocyte Subtype Analysis

#### 3.6.1. T Lymphocyte Subtype Analysis

**Figure 5:**
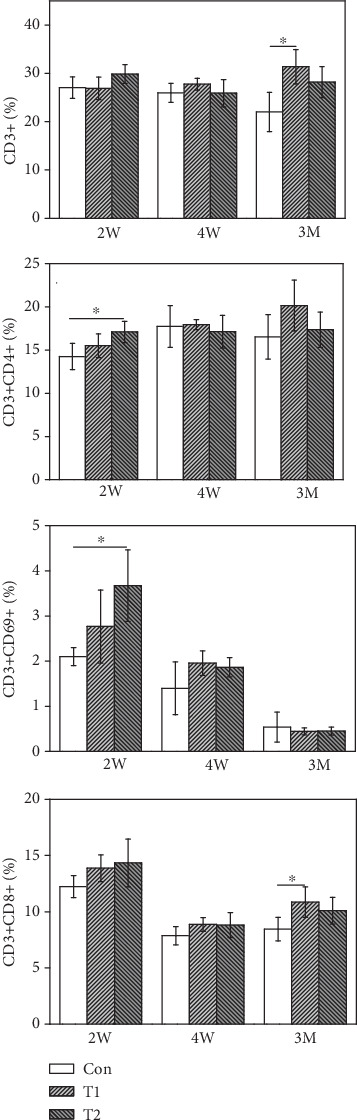
The expression of T lymphocyte subtype from T1, T2, and control (^∗^*p* < 0.05).

As shown in Figure 5, CD3+ and CD3+CD8+ T lymphocyte levels were not significantly different among the three groups at 2 W and 4 W. CD3+ and CD3+CD8+ T lymphocyte numbers of mice in the T1 group were markedly higher than those in the control group at 3 M postimplantation while no differences were observed between the T2 and control groups. CD3+CD4+ and CD3+CD69+ T lymphocyte levels of mice in the T2 group were significantly higher than those of control mice at 2 W postimplantation and reduced to normal levels at 4 W and 3 M postimplantation.

#### 3.6.2. B Lymphocyte Subtype Analysis

**Figure 6:**
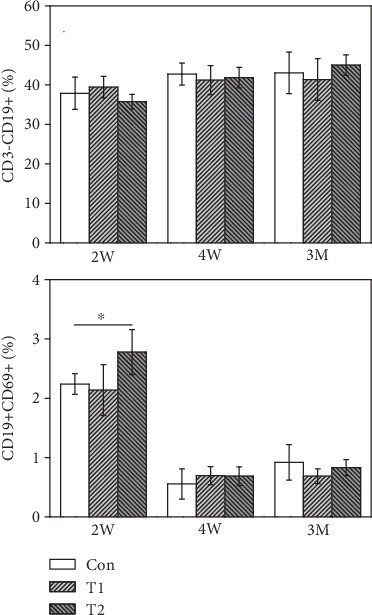
The expression of B lymphocyte subtype from T1, T2, and control (^∗^*p* < 0.05).

CD3-CD19+ B lymphocyte levels were comparable among the three groups at all implantation periods (Figure 6). The CD19+CD69+ B lymphocyte content in mice of the T2 group was significantly higher than that in control and reduced to normal levels at 4 W and 3 M postimplantation.

#### 3.6.3. NK Lymphocyte Subtype Analysis

**Figure 7:**
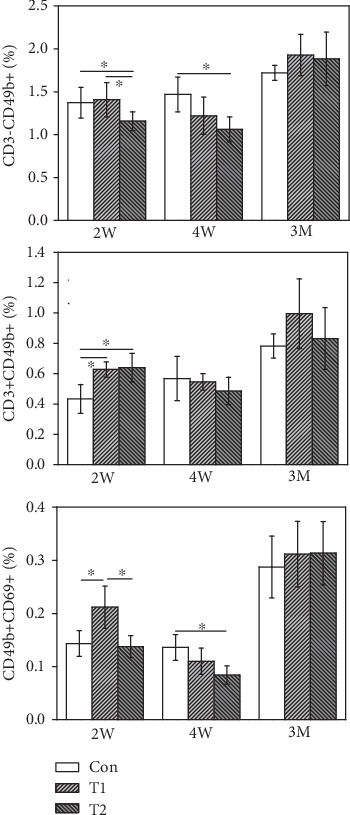
The expression of NK lymphocyte subtype from T1, T2, and control (^∗^*p* < 0.05).

As shown in Figure 7, the CD3-CD49b+ NK lymphocyte number in the mice of the T2 group was significantly lower than that in control mice at 2 W and 4 W postimplantation but recovered to normal levels at 3 M postimplantation. Levels of CD3-CD49b+ NK lymphocytes in the T1 group were not markedly different from the control group during all three implantation periods. CD3+CD49b+ NK lymphocyte contents in mice of both the T1 and T2 groups were significantly higher than that in control mice at 2 W but reduced to normal levels at 4 W and 3 M postimplantation. CD49b+CD69+ NK lymphocyte levels in T1 mice were significantly higher than those in control mice at 2 W postimplantation but reduced to normal levels at 4 W and 3 M postimplantation. CD49b+CD69+NK lymphocyte contents of T2 were markedly lower than those in control mice at 4 W postimplantation but recovered to normal levels at 3 M postimplantation.

### 3.7. HE Staining Observation of Thymus and Implanted Local Tissue

**Figure 8:**
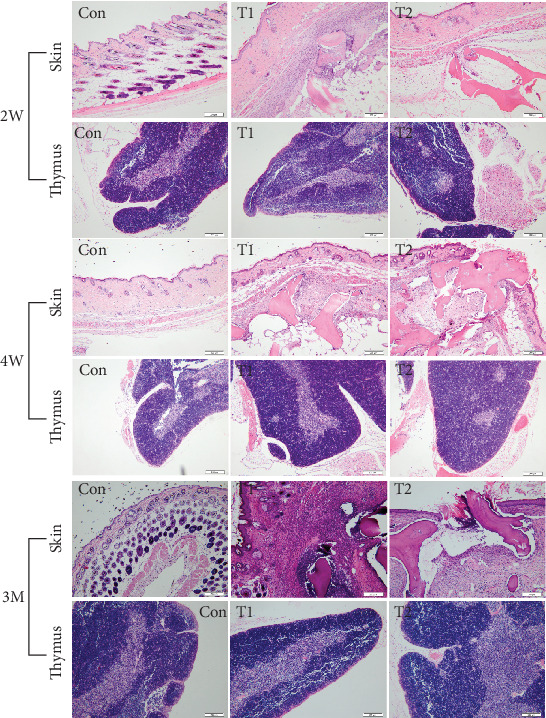
The HE staining results of the skin and thymus at 2 W, 4 W, and 3 M among T1, T2, and control (scale bar = 200 *μ*m).

HE staining results of implants and thymus at 2 W, 4 W, and 3 M postimplantation are shown in Figure 8. During the experimental period, the thymus in all three groups displayed no pathological changes. At 2 W postimplantation, a cyst with red osteoid tissue and bone lacuna was clearly observed in subcutaneous tissue (surrounding implants) of mice in the T1 group. The cyst wall comprised fibroblasts and collagen fibers. However, no nuclei were observed in the bone lacuna. T1 bone substitutes had a crumbling appearance surrounded by fibroblasts and inflammatory cells, including macrophages and neutrophils. In the T2 group, bone lacuna contained more osteoid tissues than the T1 group and a lower number of inflammatory cells surrounded the implants. At 4 W postimplantation, pathology in the T1 and T2 groups associated with the inflammatory response was similar to that at 2 W. After 3 M postimplantation, the T1 and T2 groups exhibited no significant changes, compared to 2 W and 4 W groups, other than a slight decrease in inflammation.

## 4. Discussion

In present study, to identify a suitable animal model for immune risk assessment of animal-derived biomaterials, G/i DKO mice were generated, in which the binding of anti-Gal antibody to the *α*-Gal epitope is the main cause of undesirable immunological responses [23].

Firstly, to detect the expression of *α*-Gal epitope, the *α*-Gal epitope expression profile of the novel G/i DKO mouse model was confirmed by a standardized ELISA inhibition assay. The results showed that in selected tissues of G/i DKO mice, *α*-Gal epitope expression was decreased by 100% relative to WT mice, clearly indicating that double knockout of *GGTA1* and *iGb3S* genes leads to complete elimination of *α*-Gal epitope expression. To our knowledge, this is the first report to provide direct evidence that *α*-Gal epitope expression is attributable to both *GGTA1* and *iGb3S* genes.

Previous studies have shown that expression of the *α*-Gal epitope is significantly decreased but still detectable in *GGTA1* KO animals, such as Fayez et al.'s that reported that a humanized mouse model which is lacking *α*-Gal epitope gene could still detect low levels of the human anti-Gal antibody, leading to the suggestion that *iGb3S* may be another contributory gene to Gal epitope expression [19, 24–26]. *iGb3S* gene knockout induced an ~5.19–21.74% decrease in *α*-Gal epitope expression of *iGb3S* by our group [20]. Interestingly, *α*-Gal epitope expression in the main organs of *GGTA1* KO mice is decreased by about 97–99%, compared with that in WT mice (our unpublished data). Therefore, G/i DKO mice may present a more sensitive model than the *GGTA1* KO mice for immune risk assessment of animal-derived biomaterials.

Secondly, to investigate immunological responses to xenoimmunogen, G/i DKO mice were immunized with RRBC (expressing *α*-Gal antigens). Anti-Gal antibody titers in G/i DKO mice were significantly increased from 80- to 180-fold, indicating the prominent sensitivity of G/i DKO mice to the stimulation with *α*-Gal antigen-positive material. LaTemple et al. reported 32-fold increased anti-Gal IgG titer of five week-old alpha 1,3GT (*GGTA1*) KO mice after immunization with RRBC [27]. Consequently, this result showed that G/i DKO mice were considerable sensitive to the RRBC stimulation.

A reasonable animal model is one of the important prerequisites for an effective analysis of immunogenicity in xenotransplantation research [28]. Sun et al. assessed the immunotoxicity of xenogeneic bone by embedded materials into the intermuscular space of Balb/c mice was insufficiently scientific [29]. Third, to further verify the applicability of G/i DKO mice as a model for the assessment of immune responses of xenoimplants, G/i DKO mice were implanted with raw lyophilized bone substitutes (T1, containing high Gal antigens, (8.14 ± 3.17) × 10^12^/mg) and Guanhao Biotech bone substitutes (T2, ~50% decreased *α*-Gal antigen relative to T1 [17]).

Our results demonstrated that T1 stimulate a higher anti-*α*-Gal antibody expression level in G/i DKO mice compared with the T2 group, which was similar to our previous findings using *GGTA1* KO mice [17]. However, T1 implantation did not cause a significant increase in anti-Gal IgM and IgA levels in *GGTA1* KO mice [17]. Therefore, G/i DKO mice may present a more sensitive model than the *GGTA1* KO mice for evaluating xenogeneic bone implants based on anti-Gal antibody titers. Anti-*α*-Gal antibody levels in the T2 group were insignificantly variable compared with control, indicating that the lower *α*-Gal antigen content of T2 is acceptable and no *α*-Gal antigen-related immune reaction occurred after transplantation.

Monitoring the individual dynamic immune response is therefore crucial to determine the optimal degree of immune reaction required in each transplant recipient. The measurement of selected immune biomarkers can help identify patients at a high risk of rejection. Cytokines are secreted as small cell-signaling protein molecules that can mediate effector and regulatory effects on the immune response [30, 31]. Th1 cytokine-mediated cellular immunity is mainly represented by IFN-*γ*, IL-12P70, and IL-2, while Th2-mediated humoral immunity is mainly regulated by IL-4, IL-5, IL-6, and IL-10. Liang et al. demonstrated a critical role for graft-produced IL-6 in allograft rejection in a murine model of cardiac allograft transplantation [32]. Skurkovich et al. reported antihuman interferon-*γ* Fabs may be effective in halting corneal transplant rejection after penetrating keratoplasty [33]. Liang et al. showed that eight genes (IL-1*β*, IL-1RA, macrophage inflammatory protein-1 beta, monocyte chemoattractant protein-1, CC-chemokine receptor (CCR)1, CCR2, CCR5, and F4/80) become candidates for essential functions during rejection [34]. Our study showed IL-6 and IFN-*γ* expressions in the T1 group were significantly higher than those in T2 and control, indicating that IL-6 and IFN-*γ* could serve as sensitive indicators of immunogenicity of xenogeneic bone matrix. Hence, our results indirectly confirmed the chronic immune rejection induced by T1 bone substitute transplantation via IL-6 and IFN-*γ* secretion. However, IL-1*β* levels in the T1 and T2 groups were not significantly different relative to the control group after implantation. The reason for the above results may be attributed to IL-1*β* levels not detected earlier after transplantation. He et al. analyzed the serum levels of acute phase cytokine IL-1*β* increased within 24 hours after transplantation [35].

Chong et al. showed peripheral lymphocyte subset populations are altered with the immune status of the body after organ transplantation [36]. The expression of CD molecules in different lymphocytes has different significance after solid organ transplantation, such as the heart [37], liver [38], kidney [39, 40], and lung [41]. Both CD4+ and CD8+ T cells actively participate in acute rejection through the production and release of different proinflammatory cytokines; CD4+ T cells mainly mediate the rejection response, whereas activated CD8+ T cells mediate the cytotoxic response and infiltrate the graft at the time of rejection [42]. Our study showed that lymphocyte subsets induced by T1 group were recovered to normal levels at 3 M postimplantation except CD3+ and CD3+CD8+, indicating CD3+ and CD3+CD8+ could be used as an observational indicator of long-term chronic immune response. However, there was no abnormal observation of CD3+ and CD3+CD8+ expression at early implantation. Generally, the transplantation of biomaterials is not completely consistent with organ transplantation. Treatment of the xenogeneic tissue with *α*-galactosidase has been proposed to minimize potential adverse immune responses to these graft materials. Solid organs contain blood vessels and more cell components, while biomaterials only contain extracellular matrix and a few active components after decellular treatment. Therefore, when biomaterials are implanted into the body, they generally cause a long-term chronic immune response other than acute immune rejection. Biologic scaffolds composed of ECM differ markedly in the elicited host tissue remodeling response, because the ECM constituents affect the response. The nonspecific changes in lymphocyte surface molecules in T2 groups at 2 W and 4 W postimplantation may be related to early exotic stimulus but not released *α*-Gal antigen.

Detection of Ig antibody is a basic index for monitoring organ transplant rejection [43]. However, the results on serum antibody levels demonstrated no significant differences in total IgG, IgM, and IgA levels between T1 and control at the three transplantation time-points, suggesting that total antibodies are not sensitive factors for evaluating xeno-immunological response from animal tissue-derived implants. Also, the insignificant changes in HE staining examination in T1 and T2 groups were observed due to superficial irritation of implanted materials but not released *α*-Gal antigen.

Finally, this study showed *α*-Gal epitope expression was completely eliminated in the main organs of G/i DKO mice, which induced considerable sensitivity to RRBC stimulation. Then, G/i DKO mice were applied as a novel animal model to evaluate the immune risk of bone substitutes. T1 bone substitutes showed significantly higher levels of anti-*α*-Gal antibody and inflammatory factors than T2 bone substitutes. However, no significant changes can be found between the T1 and T2 groups in terms of the total antibody levels, spleen lymphocyte surface molecules, and HE staining observation. Therefore, it can be concluded that the G/i DKO mice can be used to evaluate the *α*-Gal induced immune risk of animal-derived bone substitutes.

## 5. Conclusion

In summary, G/i DKO mice are sensitive to Gal antigen-positive xenogeneic grafts and can be effectively utilized for evaluating *α*-Gal-mediated immunogenic risk of xenogeneic grafts. G/i DKO mice were more sensitive than *GGTA1* KO mice with regard to evaluating the effects of xenobone implants based on anti-Gal antibody titers. Our experiments provide original data and theoretical support for the utility of a novel sensitive G/i DKO mouse model for the immune risk assessment of animal-derived biomaterials.

## Figures and Tables

**Table 1:** *α*-Gal epitope expression in the main organs of G/i DKO mice and WT mice (*n* × 10^11^ Gal epitope/mg).

Mouse type	Spleen	Heart	Liver	Kidney	Lung
WT	245.08 ± 48.53	93.05 ± 7.17	24.91 ± 4.88	58.56 ± 17.86	312.09 ± 31.89
*GGTA1/iGb3S* DKO	0.00	0.00	0.00	0.00	0.00

## Data Availability

The data used to support the findings of this study are included within the article.
